# World’s Columbian Dental Congress—1893

**Published:** 1893-11

**Authors:** J. M. Walker


### Society Proceedings.

### World's Columbian Dental Congress,—1893.

SCIENTIFIC WORK BY SECTIONS.

Reported for the Dental Register by Mrs. J. M. Walker.

WEDNESDAY, AUGUST 16TH. THIRD DAY, GENERAL SESSION.

(*Continued from page* 516.)

The general session of the Congress was called to order on the third day of the Congress, at 12 M. by the President, Dr. Shepard.

Drs. Godon and Ronnet, of France, and Dr. Capon from Canada, were introduced as representatives of their respective countries. After brief responses from these gentlemen, a paper by Dr. W. D. Miller, Berlin, Germany, was read by Dr. A. O. Hunt. An abstract of Dr. Miller's paper follows.

The paper was entitled "Concerning Various Methods Advocated for Obviating the Necessity of Extracting Devitalized Tooth-Pulps."

Dr. Miller spoke of the comparative ease with which the pulp is removed and the root-canal filled in the incisors and cuspids, but when we extend this treatment to the bicuspids and molars, the labor and expense put it beyond the reach of the great majority of the human race, and the method is not always successful. It will consequently be a great boon if some means or method can be devised which would render unnecessary the removing of the pulp and filling the root-canals of molars.

Dr. Miller then presented briefly the methods devised by Witzel, Baume and Herbst, the latter as put forth by its author and as modified by Bodecker, and summarized their advantages and disadvantages. He said: "I have for a long time felt that the solution of the problem was to be sought for in the direction pointed out by Witzel, except that our efforts should be directed not to retaining the vitality of the root-stumps, but to preventing their subsequent decomposition, by impregnating them with a suitable antiseptic. I am convinced that the success of the impregnation method depends to a very great extent upon the character of the antiseptic employed, and upon its chemical action upon the pulp apart from its antiseptic action.

After enumerating the essential qualities necessary to such an antiseptic, he said, that as the result of over five hundred experiments he had divided the different dental antiseptics now in use into three groups.

1. Those possessing in a high degree the power of imparting antiseptic qualities to root-pulps, such as, cyanide of mercury, bichloride of mercury, diaphtherin, sulphate of copper, salicylate of mercury, oil of cinnamon, ortho-kresol, carbolic acid, trichlorphenol, chloride of zinc.

2. Those of doubtful value: Thymol, salicylic acid, eugenol, campho-phenique, hydronaphthol, A and B naphthol, acetico-tartrate of aluminum and some essential oils, resorcin, thallin, sulpho-carbolate of zinc, oil of birch, iodide of sodium, nitrate of sodium, etc.

3. Those nearly, or quite worthless: Iodoform, basic anilin coloring matters, borax, boracic acid, dermatol, europhen, chloride of lime, peroxide of hydrogen, sozoiodol salts, iodol, tincture of iodine, spirits of camphor, naphthalin, etc.

He then gave the results of a series of practical tests with the bichloride of mercury, in between four and five hundred cases.

The following formulas were given, the combinations being used in the form of small tablets:

1. Sublimate 0.01 gram; boracic acid 0.02 gram.
2. Sublimate 0.01; common salt 0.02.
3. Sublimate 0.0075 gram; thymol 0.0075 gram.
4. Sublimate 0.005 gram; thymol 0.005 gram; tannin 0.005 gram.
5. Cyanide of mercury 0.0075 gram; thymol 0.0075 gram.
6. Salicylate of mercury in the same form.

In the use of these tablets, the pulp being devitalized, the pulp-chamber thoroughly opened and cleansed, and a tablet applied and slightly crushed, with an amalgam plugger moistened with water and covered with a layer of tin or gold foil, and the amalgam or cement filling immediately inserted.

The formulas No. 1 and 2 were abandoned, severe pain following this application in about thirty per cent. of the cases.

In No. 3 the thymol prevents the sublimate being so rapidly absorbed, besides giving a greater permanency to the application by reducing the solubility. Very seldom, so far, has pain followed the use of these tablets, while experiments out of the mouth show that they still possess sufficient penetrating power.

No. 4 does not penetrate as rapidly as No. 2, and discolors the tooth more.

Of No. 6, he says: This, I think, deserving of a trial. Its sparing solubility justifies the belief that its action will be more permanent than that of sublimate. The sulphate of copper may be used in pure form, but it naturally causes serious discoloration of the tooth at the neck, and is also, I fear, too soluble to give permanent results in pure form. More recently I have directed my experiments towards the discovery of some substance which possesses the desired qualities without discoloring the tooth. Thus far I have obtained the best results from diaphtherin (oxychinaseptol), an antiseptic recently introduced by Emmerich. It may be applied in pure form. Among liquid antiseptics the oil of cinnamon takes the first place, and I have much faith in its power to conserve the dead pulp. Like all the liquids, however, it is difficult to apply, and has, besides, the disagreeable quality of discoloring the tooth yellowish-brown. The combination which I have chiefly employed is that of sublimate and thymol. I have not had the opportunity to sufficiently test the others in practice, though I am now using, by way of experiment, the salicylate, and to some extent, the cyanide of mercury. It has been employed at the Dental Institute of the University of Berlin in over two hundred cases. Of these only one failure has come to my knowledge.

Dr. Frank Abbott gave his method of treating teeth with dead pulps as follows: He said, I never depend upon the application of an antiseptic in the roots of teeth, but upon a material which I force in and around such, with which is combined an antiseptic strong enough to answer the purpose and virtually mummify all the material that is left in the canals of the tooth by its action. It surrounds and covers it over, and whatever portion of the pulp is left behind is penetrated by the action of the chloride of zinc and bichloride of mercury that is mixed with it. Of course, if the pulps die, they die of their own accord. I have many dead teeth to handle and many to treat in my practice, as every one has who is in full practice, and I treat them all in one general way. That way is to open the pulp-chamber as carefully as I can, so that I may cleanse it thoroughly of every particle and get thoroughly into all the root-canals. I, then, with a very fine gold-pointed syringe, use a 1 in 10,000 solution of bichloride of mercury—a grain of bichloride of mercury in twenty ounces of water—and syringe out these canals just as thoroughly as I can; I, then, with a broach or small instrument penetrate into the canal as far as I can go, stir up the contents, and then wash again, repeating this until I am pretty sure that everything is clean, so that the substance coming out of the tooth as it strikes a white napkin will show a white, clean color instead of staining, as when the canal is filled with dead material. When it is washed thoroughly clean I fill with oxychloride of zinc, in which I put a drop of a solution of 1 in 2,000 of bichloride of mercury and the penetrating and antiseptic properties of the chloride of zinc and oxide of zinc.

This is the material that mummifies or holds this substance that is left in the roots of the teeth, leaving it in a condition to give no trouble; and it may astonish some of you to know that instead of opening a tooth and treating it day after day for a week or more, I open a tooth and fill it at the same sitting always, unless I have periosteal irritation—soreness of the tooth as I touch it. The crown of the tooth is filled with gold or any substance that I choose to use, and I dismiss the patient after painting the gums carefully over with a solution of concentrated tincture of aconite root and tincture of iodine. That I always do before my patient leaves the chair. It is a powerful counter-irritant, and does the work of relieving the pressure around the root of the tooth. This, to me is the simplest, easiest and most quiet way of getting along with that kind of teeth.

Dr. Geo. Cunningham, England, said that he had investigated the Herbst Method of Treatment in these cases but did not approve of it, he said: If we could find for poor people some means which would shorten the treatment, I trust it will be the practice as used by Dr. Abbott, which will give the opportunity to fill at one sitting. I think the paper we have had to-day is of very great importance, because it has pointed out one way that we can bring our operations within the reach of larger numbers of the community.

Dr. Schreier, of Vienna, addressed the Congress in German, translated by Dr. Ottofy, as follows:

It is indifferent what antiseptic is used; each one leads to the same result. It is only necessary to find material which is easily applicable. If any one says that he can take an antiseptic material and inject it into the fine canals, it is a matter which is impossible to comprehend. It is necessary that the material should be one which is readily introduced into the root-canal, and the effect of which is prompt and immediate. Such a material Dr. Miller did not mention in his essay, but I have published an account of Potassium-Sodium, which, on another occasion, I will present to the members of the Congress.

The paper was then referred to Section 99, "Etiology, Pathology and Bacteriology," for further discussion.

Various announcements were made, and the General Session adjourned till Thursday at 12 M.

### WORK OF THE SECTIONS, WEDNESDAY, AUGUST 15.

### SECTION 1.—ANATOMY AND HISTOLOGY.

The Section was called to order by Dr. R. R. Andrews, Chairman.

Dr. S. H. Guilford, of Philadelphia, Pa., read a paper entitled "The Teeth and Hair; Their Homology and Pathological Intimacy."

He said: Perhaps no organs of the human body exhibit such a variety of aberration from the normal type and number as the teeth, and this fact further furnishes a field for observation and study. The subject is broadened and the interest intensified when we consider the teeth in relation to other organs or structures with which they are intimately associated.

Of the epithelial products the hair, next to the teeth, is the one that is most frequently found to be abnormally affected, and the fact that these two structures have often been found to be conjointly influenced long ago attracted the attention of scientific observers, but seems never to have received the careful investigation that its importance demands.

He then made a study of the origin of these structures, and cited a large number of interesting and striking cases, from all of which he drew the following conclusions :

First. That where one product of the epithelial layer is abnormally affected one or more of the other products of the same layer are also apt to be.

Second. That the two most commonly affected are the hair and the teeth.

Third. That this is shown by numerous examples throughout the vertebrata, including man.

Fourth. That the intimacy between these two products is probably due to the contemporaneousness of their inception, as well as to many points of similarity in their character and growth.

Fifth. That when thus co-ordinately affected the manifestations are not uniform, but variable.

Sixth. That this variability cannot be accounted for in the light of present knowledge.

In the discussion of this paper Dr. Thompson, Topeka, said : There has been such a limited amount of observation and record in regard to this complicated subject that very little can be said about the law that may govern the correlated variation, and nothing can be said as to the real law. Darwin was, perhaps, the first to notice the variation and abnormality of teeth and hair together. Of course, the teeth and hair, both being dermal structures, are liable to be varied together where there is any abnormality of development, whether pathological or not. There is something peculiar about that to which Dr. Guilford has referred. While we call teeth dermal structures we know that they are not entirely so. The dentine is certainly an osseous structure, so that it is quite a question whether we are justified in calling the teeth, as a whole, dermal structures.

Dr. Sudduth cited a case in which there was also a lack of development of the sudorific glands. He said: This case evidently was one where the fault lay in some nutritive disturbance about the sixth week of intra-uterine life, when the hair and teeth begin their development; the individual after arriving at maturity had overcome this disturbance and developed a second set of teeth normally. The case was plainly one of nutritive disturbance at that time. We have not enough cases on record to formulate any law regarding this peculiar phase of abnormal development. There is no regularity in the disturbance of the hair and teeth. In some of the cases cited the teeth were fairly normal and the hair was abnormal; in others, the hair was normal and the teeth abnormal. There can be no question about the homologous relation between the hair and teeth. I think there was a time in the history of the evolution of the teeth when they were not as perfect as at present, when they could have been classified more nearly as scales or dermal appendages.

Dr. Frank Abbott, New York: I do not understand that the teeth as such are of dermal epithelial production. It is the enamel of the tooth that is of epithelial production, the other portions being of a different structure and from a different source.

Dr. A. H. Thompson, Topeka, Kansas: In the lower animals the teeth are entirely osseous, and quite identical with the bones of the skeleton and jaw. As we advance in the scale of development the enamel is added to the osseous structure or dentine.

Dr. Sudduth: In the development of teeth the development of the enamel-organ must invariably precede the osseous. The history of the teeth and the hair is identical, both beginning at the same time—the sixth week of intra-uterine life. The epithelial buds must be there both for the hair and the teeth.

Dr. Frank Abbott, New York, then read a paper entitled, "Teeth of the Lower Jaw at Birth."

This was a continuation of the studies of Heitzmann, and Bodecker in the development of teeth during the last months of fœtal life, for which purpose the lower jaws of apparently well-developed, new-born babes were excised soon after death, stripped of their soft tissue, and placed for preservation in alcohol. Afterward they were placed in a one-half of one per cent. solution of chromic acid, for the purpose of decalcifying the hard tissues, and, at the same time, to preserve the soft structures.

The further process of preparation of the specimens was minutely described, and the stage of development of each tooth clearly illustrated and described. He regarded these specimens of special value, as showing the space between the inner epithelium and the already-formed tooth, produced by a detachment of the former from the latter, which plainly shows a layer of protoplasmic bodies into which the ameloblasts have retrogressed before becoming infiltrated with lime salts. Since this feature is pronounced in all the teeth of the jaws under consideration, the specimens are of great value in assisting, at least, in settling the still mooted question as to the mode of development of the enamel. This was especially true of the section, obtained from the cuspids, which, he said, were so perfect that they could be utilized advantageously to assist in settling certain mooted questions as to the development of dentine and enamel. In the first place, the odontoblasts have, since their discovery, been considered by the majority of histologists as the dentine-formers proper.

It was Heitzmann and Bodecker, in their above quoted article, who first denied the direct transformation of odontoblasts into dentine, but claimed that they are first broken up into medullary corpuscles, at their distal ends, which become infiltrated, first, with a glue-yielding basis-substance, and afterward, with lime salts, and that the offshoots of the original odontoblasts, being the dentinal fibrillæ, ran between the calcified medullary corpuscles in their respective canaliculi. Every odontoblast sends off one or more fibrillæ. This fact, with some observers, renders the formation of the basis-substance not clearly understood. So great indeed has been the difficulty that E. Klein, of

London, and R. R. Andrews, of Cambridge, have resorted to specific fiber-cells for the production of these fibrillæ, whilst the odontoblasts proper produce the basis-substance.

This view, he continued, was met at the time by a demonstration showing that the "fiber-cells" were wedge-shaped odontoblasts, and most numerous where the odontoblasts are arranged in a sharply-curved line, especially at the summit of the papilla. In the pig's fœtus, for instance, the summit of the papilla is occupied almost exclusively by such narrow and wedge-shaped odontoblasts. The difficulty is, however, easily overcome by the demonstration of medullary corpuscles at the periphery of the papilla, directly beneath the already-formed dentine. In my specimens, especially the cuspid, one is struck by the scantiness of rows of odontoblasts, and the presence of medullary elements in their stead; one row being visible on a portion of the labial, another on a portion of the lingual surfaces only. Not infrequently the odontoblasts are arranged at acute angles to the dentinal canaliculi. The greater area of surface of the papilla is occupied by a medullary tissue, claimed to be the dentine-former proper. In our cuspid the summit is occupied by a myxomatous tissue, approaching in gracefulness almost that of the enamel-organ. Between this myxomatous tissue and the border of the non-calcified dentine clusters of indifferent or medullary corpuscles are seen, arranged longitudinally along the surface of the papilla, therefore, not as yet arranged for the formation of dentine. Beneath the myxomatous tissue we find vascularized myxo-fibrous tissue, constituting the main bulk of the papilla.

Obviously there is a series of tissue changes preceding the appearance of dentine, and one of the links in the chain is the odontoblast. At the lateral portion of the cuspid the question of the formation of enamel may possibly be settled. The same elements of proof of the position taken are traced through the appearance presented by the permanent cuspid and the first temporary molars.

The first permanent bicuspid, the product of the bud from the first temporary molar, at birth corresponds to a temporary tooth four and a half months old. It is cone-shaped and composed of medullary tissue, with but scanty capillary blood-vessels at the base of the cone. As a matter of course there is not even a trace of odontoblasts visible. The papilla is covered with a layer of columnar epithelia, the so-called inner epithelium of the enamel organ, not as yet transformed into ameloblasts. The point of recuravation of the inner into the outer epithelium is deeper down upon the buccal than upon the lingual aspect of the papilla. The outer epithelium is still recognizable as being composed of short columnar epithelia, surrounded by fibrous connective tissue. Between the inner and the outer epithelium there is a well-developed myxomatous reticulum, the *enamel-organ.* The intermediate layer is present, though, as yet, little pronounced.

In conclusion, he said: Heitzmann and Bodecker, as the result of eight years' hard labor, came to the conclusion that the elements of the original epithelium are reduced to medullary tissue, afterward to fibrous connective-tissue, and eventually to ameloblasts. Ameloblasts, according to their notion, are not direct enamel-formers, any more than odontoblasts are direct dentine-formers. The ameloblasts are reduced to medullary corpuscles, which after infiltration with the basis-substance and immediate deposition of lime salts, at last produce enamel-prisms. From a study of their specimens and my own I am convinced that these views are correct.

This paper was discussed by Drs. R. R. Andrews, W. X. Sudduth and A. H. Thompson, the result being thus summed up by Dr. Sudduth:

This subject has been gone over for years, and I do not think we will ever be able to come to an understanding of the true interpretation of the phenomena. I do not see that we are any nearer than we were in 1885, when we first began to discuss this subject.

Dr. Sudduth then announced that the regular programme for the evening would be the presentation of Dr. Andrews' paper on the "Development of Enamel," virtually a continuation of this subject, and his own paper on "The Forces that Tend to Modify the Form of Teeth and Jaw."

Adjourned until Thursday afternoon at 2:30 o'clock.

SECTION 2.—ETIOLOGY, PATHOLOGY AND BACTERIOLOGY.

In the absence of the Chairman, Dr. G. V. Black, Dr. J. D. Patterson took the chair, and appointed Dr. Davis as Secretary, Dr. Chisholm being absent. No discussion was had on the subject of the General Session referred to this Section.

Dr. L. C. Ingersoll read a paper entitled, "The Relation of Predisposing Causes [so-called] to the Active Causes of Dental Decay," of which we give a brief abstract:

Dental Pathology presents one aspect that at once arrests the attention of mankind, and which is made the basis upon which the dental profession is built, viz.: The fact of dental decay, without which the dental profession would never have come into existence. The subject of the etiology of dental decay is commonly presented under two heads—*existing* and *predisposing* causes. The use of the term *predisposing* causes is open to criticism. The relation of causation to results is that of effective means acting to produce the results. Mere passivity cannot be a cause, for a cause implies activity. Mere presence and passivity may be conditions affecting the operation of active causes, and such is the view that should be taken of what are commonly known as *predisposing* causes of dental decay.

A comprehensive view of all pre-existing conditions in this connection, he said, would embrace a complete history of an individual, reaching back over many ancestral lines, inquiring into inherited idiosyncrasies, and the effect of every extraneous influence that could in any way enter as a factor into tissue and organic development. This cannot be attempted here. It is sufficient to say that every tissue of the body, whatever its general law of physiological development, has come to be what it is through various modifications of its typical nature, and has received an impress peculiarly its own, derived from a great variety of sources.

Our object is to learn, if possible, to what extent pre-existing conditions are responsible for the exciting or active causes of decay. It was a great step forward in accounting for dental decay when, what had long been a mere presumption became a demonstrated fact, that micro-organisms are a potent factor in breaking down the hard tooth structures. The influence of the teaching was so widespread and so deeply impressed, that many became ready to renounce all other theories and demonstrations touching the subject, forgetting for the time being the conditions that restrain the active working of bacteria.

There seems to be an abiding impression in the minds of some that if bacteria are found in the mouth they must exercise a destructive agency upon the teeth, and that prophylactic antisepsis is absolutely essential to prevent decay.

The chemical theory of the decomposition of the mineral portions of teeth must be accepted from first to last; but why should it be thought necessary to abandon a theory of decay by vegetable and mineral acids chemically produced, when accepting the theory of decay by acids produced by organic germs? It is chemical decomposition in either case, with the additional destructive work of the bacteria gaining their food-supply by devouring a portion of the organic matters composing, in part, the substance of the teeth.

A number of the so-called predisposing causes of decay were next considered by the essayist, leading to the conclusion that the etiology of dental decay is not to be found alone in external influences, but also in the inherent nature of tooth substance, as, for instance, in the face of the teaching that if teeth are only kept clean they will not decay. We find teeth that are kept clean to the most extreme limit of practicability, decay early and rapidly; and, on the other hand, we find teeth that are never cleaned from month to month, retain their firmness of substance to a good old age, in spite of the microbe.

When the favoring and restraining conditions of dental decay are compared, it will be found that the latter far out-number the former, and are far more potent in their influence.

Were it not for the restraints and antagonism set up in the mouths of the human family, every tooth would melt down under the influence of decay within a year, and not one be seen in human jaws; and that, too, in spite of every practicable system of antisepticism that could be adopted. Antisepticism is not a cure; prophylaxis is.

Here there meets us the grandest theme ever presented in dental literature, the prevention of dental decay. Not that prevention that arrests decay when its work of destruction is half done, but that prevention that does not allow the work of decay to begin; that prevention which establishes by hygienic law a barrier in the very nature of tooth-substance that will effectually resist all external influences; that prevention that reaches back to embryonic life, and touches the protoplasmic germ, and stamps it with longevity. We need a better understanding of that dental hygiene that guards the very portals of life and nutrition, and forbids the entrance into the tissues of the teeth of every element of weakness and decay.

The paper was discussed by Drs. Moore, of California, J. W. Morrison, of Nashville, Tenn., E. S. Chisholm, of Alabama, and L. L. Davis, of Chicago.

In concluding the discussion, Dr. Davis said that he understood so well Dr. Ingersoll's position in regard to bacteriology that he did not for a moment intend to detract from it. He was simply calling attention to the fact that possibly some of us have been led a little astray in the study of bugs. As one gentleman has already said "we should look for the highest development of the species rather than go into the theory so far that we forget even the prominent points that must be ever kept in mind."

A paper on "Oral Pathology," by Dr. R. Finley Hunt, Washington, D. C., was read by the Secretary of the Section.

The paper was a brief treatise on but one of the many pathological conditions to which the oral organs are subject. It is that pathological condition of the oral mucous membrane and subjacent tissues commonly known as the "Rubber Disease," or "Rubber Sore Mouth."

Though quite a number of dentists deny the existence of this disease as being produced by the wearing of rubber plates, *per se*, yet the concordant testimony of members of the profession of the last and the present generation seems to establish beyond controversy not only its existence, but its existence as a specific disease produced by a specific cause, viz: the wearing of rubber plates in direct contact with the mucous membrane.

This testimony obtained from the records of dental societies and associations, and from dental journals commencing about the year 1866, i.e., soon after dental rubber plates came into use, identifies the pathological condition now under examination as a specific disease with distinct symptoms and characteristics, and establishes beyond controversy, the fact that it is caused by wearing vulcanized rubber in direct, constant, and adhesive contact with the mucous membrane in the mouth. From the same evidence may be deduced its symptoms, stages and effects, as follows: A low order of vitality in the parts covered by the plates; a peculiar pallor of the same, irritation, swelling, redness, inflammation, congested, engorged, hyperemic condition; granulated masses like a strawberry, red, purple, scarlet, soft and spongy; half the arch filled with a spongy mass; blood oozing from the diseased parts; turgidity of the vessels; roof of the mouth like half-decayed raw beef cut across the grain; suppurating, discharging condition, ulceration; pus exuding from the folds; sanguino-purulent fluid constantly exuding from the apertures in the palate over the necrosed bone; sensitiveness so obtunded that no pain is felt; or in other cases, a burning, drawing, feverish sensation, lines of inflammation extending to the throat, causing a disagreeable tickling sensation, and annoying cough; bronchial affections, chronic catarrh; sloughing of the soft parts; in some cases death.

A cure may be effected, and a recurrence of the disease prevented in three ways, viz.: By discarding altogether the rubber plate; by substituting for it a plate of gold, platinum, or even silver; and by interposing permanently between the rubber and mucous membrane a foil or thin sheet of metal.

The range of metals that have been found successful in relieving and curing this disease is wide—as gold, platinum, silver, tin, and aluminum. The requisites most effective in the foil used are, as shown by experience—purity, cohesiveness, and a permanent union with the rubber. Gold, as at present prepared, best meets these requirements.

The subject was discussed at considerable length by Drs. Noble, Washington, D. C.; Walker, Bay St. Louis, Miss.; H. C. Miller, Oregon; I. P. Wilson, Burlington, Ia.; J. D. Patterson, Kansas City, Mo.; Cook, Moore, Nelson, McMillen, G. B. Huff, Chisholm and Boyd, Alabama.

The evils admitted from the use of rubber plates were variously attributed to ill-fit, lack of cleanliness, mercurial poisoning from the coloring matter of red rubber, the non-conducting character of the material, etc.

At the close of the discussion the section adjourned till Thursday afternoon.

### SECTION 3.—CHEMISTRY AND METALLURGY.

This Section held no meeting.

### SECTION 4.—THERAPEUTICS AND MATERIA MEDICA.

Meeting called to order at 2:30 P. M., Dr. F. J. S. Gorgas, in the chair.

The discussion of the subject of "Local Anæsthesia by the Use of Cocaine" was continued by Drs. Hewitt, M. L. Rhein, J. G. Templeton, Geo. A. Mills, J. S. Lowry, Bigelow, N. S. Hoff, A. C. Hart, Cravens and Horton.

Dr. Hewitt said: I advocate the use of cocaine daily in every dental office. Every time that you pass a gilling twine around a tooth to carry the rubber-dam up high enough to pass the cavities, you inflict pain. You have no right to do that. Whenever you put a rubber-dam clamp on a tooth without first obtunding the sensitive dentine, you are negligent of the first duty of the dentist to his patrons.

There are ways in which painlessness may be acquired in burring sensitive dentine, in cutting down on to the live pulp, in amputating the crown of the live tooth, in amputating exostosis of the jaw, in removing a tumor that may be situated upon the tongue, in performing any operation upon the mouth with the patient perfectly conscious, able to open his mouth upon request, to spit out the blood when it is required, and absolutely without pain.

He then gave the following formula, for local application, which he has named: "Compound Cocaine Pigment."

Atropin, one-tenth of a grain.

Stropanthin, one-fifth of a grain.

Hydrochlorate of Cocaine, one hundred and twenty grains.
Hydrate of Naphthol, ten grains.
Chloroform, two drachms.
Oil of Cassia, two drachms.
Glycerine, one drachm.
Atropin, to guard against the toxic effects of the cocaine.
Stropanthin, the best known heart-tonic in existence.
Naphthol, as an antiseptic.

The glycerine is simply a solvent to hold in solution the one hundred and twenty grains of hydrate of cocaine together with the oils of the mixture, preventing precipitation.

Dr. Rhein protested vigorously against the recommendation of the universal use of cocaine, a dangerous toxic drug, and said: I feel sorry for the gentleman that can't adjust a rubber-dam clamp painlessly. The application of cocaine is useless unless it is introduced hypodermically. A mere application of the drug superficially on the mucous membrane, beyond the faith which you may instill into the patient, or the delicate manner of your manipulation, will do no good. For that class of neurasthenic patients whose system is not anemic, whose hearts are in that condition that they can stand the use of cocaine, the careful injection, hypodermically, of a good amount of cocaine, the hydrochlorate, before putting a clamp very deeply, or before the immediate separation of the teeth by a separator, or in the extraction of a live pulp or a root, is a very valuable adjunct to dentistry, and one that can be recommended to every man who has sufficient knowledge of therapeutics to be enabled to know the proper drug that ought to be used as an antidote, or to take care of his patient if any ill effects follow.

Dr. Templeton described some cases in which he had seen very good results from Dr. Hewitt's use of his "Pigment." He said, that the operator first applied to the gums a solution of chloro-hydrate and naphthol, for the purpose of absorbing and washing away the mucus on the gums, so that the pigment would take effect, and after an application of the cocaine preparation, the application of the rubber-dam produced no pain.

Dr. Lowry said: One point that I think is one of the most important features of hypodermic injection of cocaine, is, never inject cocaine into the muscular tissue; if you do, it is carrried into the circulation *en masse* to the heart, and you produce heart-excitation and sometimes nausea. But if you inject the cocaine into the hard gum-tissue, being very careful that your needle is so located as to diffuse the cocaine slowly, in such a way that you can see the gum whitening gradually, and the white surface gradually enlarging, you will rarely ever have nausea.

Dr. Bigelow uses cocaine upon the gingival margin in the application of the rubber-dam in very difficult cavities, applied by means of a very small pledget of cotton dampened with water, sometimes with alcohol, dipped in the pulverized cocaine and applied to the gingival margin.

Dr. N. S. Hoff said: I do not believe in using prescriptions containing supposed cocaine antidotes, such as we have had presented here to-day. This prescription makes, as I figured, a twenty per cent. solution of cocaine, or more. The effects of cocaine are two—general and local. It is the local effect that we want, and not the general effect. But this prescription provides for counteracting the toxic effects of the cocaine; it pretends to make provision against that in the use of sulphate of atropia and stropanthin; these are both heart stimulants, or rather they stimulate the nerves that control the action of the heart. But the doses are too small for the amount of cocaine that is used. This is a dangerous prescription, even used locally on the gums. You all know the histological structure of the gum-tissue, how slowly it will take up medicine; it absorbs slowly, yet at the same time it will absorb large percentages of cocaine preparations, especially when such agents as chloroform and glycerine are used in connection with them. The cocaine will be taken into the circulation, and readily brought to the nerve centers, and toxic effects will be produced. There is nothing at all in this prescription that is corrective. There is nothing that will hinder the dissipation of this drug, or limit its dissipation in the tissues to which it is applied, and it will be taken up by the blood-circulation of the gums and carried directly into the blood-circulation throughout the entire system.

Dr. Rhein: I want to correct a wrong impression which my words seem to have left. I do not question for a minute the ability to produce local anæsthesia by any such prescription as that. The object of my remarks was to disparage the universal use of cocaine, and I say that any such doctrine as that is damnable. I never intended to leave the impression that I disparaged the possibility of its producing the results. I am in the same position with the gentleman who said he had used cocaine for six years. I have used it since it was first brought into New York City, and I use it every day; and I use it as he says he uses it. I prepare a fresh solution with distilled water, and only use it during that day, and I believe it is the safest way. But I use it with discrimination—that is the point I wish to impress. The gentleman stated that he used it universally, and that is the danger-point in any such advice.

A spirited dialogue between Drs. Mills and Rhein followed these remarks.

Dr. A. C. Hart, San Francisco: There has been a great deal said upon the subject of cocaine, and some of us who are young in practice would be confused and go home from this convention and not know what to do. Really nothing has been said as to what cocaine is, or how it acts.

Dr. Hewitt: In reply to my friend, Dr. Rhein, when I suggested the use of cocaine, I did it advisedly, knowing I was talking to men of education, of refinement and careful thought. Have I got to go into the minutiæ, and tell them that they must not give a neurasthenic patient cocaine, or a fat man might be an improper subject to use it upon, or that a delicate woman shall not have it, or a little child, and so on? I took it for granted that these men know something. I had only a few minutes, Mr. Chairman, in which to present this. I would like if I had time, to tell you how I use it, and I defy my young friend to prove that the application of it is damnable. I ask you as humanitarians, as men of science, to think of this, read upon the subject, and write to me and ask any questions that you please, and I will answer them with the greatest of pleasure.

On motion, the paper of Hedwig Bensow Stahlberg, of Helsingfors, Finland, on "Ethyl Chloride as a Local Anæsthetic," was read by the Chairman.

I desire to offer a few suggestions in relation to an anæsthetic that has recently been highly spoken of in our profession.

After a brief review of authorities on the use of this agent, the writer continued: In the use of ethyl chloride, I apply it directly on the gum and to the immediate neighborhood of the root or roots of the tooth to be extracted. I cover the other teeth in the same jaw, and the inside of the mouth, as well as the tongue, with prepared cotton, because I find that the cotton lies more closely to the surrounding parts than a napkin, as proposed by Professor Carlson.

This cotton covering should be carefully made, so that it at the same time prevents the saliva reaching the locality to be anæsthetized, and also prevents the patient from swallowing any of the agent. Just as in using other local anæsthetic agents, the time required to secure insensibility is very variable. Some patients require only about one gram; others three or four grams, and exceptionally even more. In one case I found that ten grams were necessary. These circumstances, however, are unimportant, because the agent is very harmless, and it is so cheap the poorest patient can afford to have it used.

At the conclusion of this paper, the paper read by Dr. W. C. Davis on Tuesday (see page 500, October issue), was discussed in connection with the paper on "Ethyl Chloride."

Dr. Lowry said: I want to speak of one point made by Dr. Davis, of Nebraska, which I think is a very good one, that the tubuli of the dentine are not occupied by a nerve-filament; the nerve must be nourished by blood-circulation. The tubuli of the dentine are too small for the free circulation of the red blood-corpuscles, the red corpuscles being three-ten-thousandths of an inch in diameter, and the tubuli only one-ten-thousandth of an inch or one-third the size. Consequently, there can be no blood-nutrition to that nerve. Secondly, he says, it is a protoplasm. I think that is a good point. The tubuli are occupied by protoplasm. His idea is to apply warm alcohol to the cavity of the tooth and by its evaporation extract the protoplasm, thereby disposing of the conducting medium or the medium that conducts the sensation from your instrument to the nerve or the pulp proper.

I have used warm alcohol for probably eighteen months or two years, almost to the exclusion of anything else, for obtunding the sensitive dentine. I knew it was of benefit, though I did not know why, but this idea of the essayist has brought it clearly to my mind what the alcohol did accomplish, by its action upon the dentine protoplasm.

So far as the chloride of ethyl mentioned in the last paper is concerned in its application for the obtunding of sensitive dentine, I think it is the most painful thing that was ever administered or put into a cavity. I do not think its effect is so painful upon the gum, but to place it within a cavity for the obtunding of sensitive dentine it will inflict more pain than the excavation of the cavity without the use of an obtundent.

Dr. Rhein spoke of the superiority of chloride of methyl over the chloride of ethyl. He said: Chloride of ethyl is so different from the chloride of methyl that it takes several minutes in the use of chloride of ethyl to produce by its evaporation a reduction of temperature equivalent to between zero and ten degrees above zero, Fahrenheit, while chloride of methyl by its greater volatility, in the course of five seconds will produce a reduction of temperature equivalent to seventy degrees below zero—it will freeze the mercury in the bulb of the thermometer in five seconds. Now, the danger of it is the possible effect on the pulp of the tooth, and it must not be overlooked. In all the length of time I have used it, I have not, to my knowledge, had any bad effect on the pulp, apart from some slight irritation of the pulp following in some cases where I was impelled, more through a feeling of investigation than otherwise, to use it in a deeper cavity than I would use it to-day; but in those very sensitive labial and buccal cavities, where we really find the greatest difficulty in commencing to excavate, or where we want to build up a tooth that is very sensitive, in fact all cavities that are remote from the pulp, the most infinitesimal amount of the fluid sprayed from the cylinder direct on the tooth will produce an immediate insensibility of the part, if it is used with some little intelligence, but all you require is a very small effect. If you allow it to come out in a minute stream in the way in which the chloride of ethyl comes out, you can entirely remove the pulp from the tooth by its means.

A paper by Dr. D. Caracatsanis, of Athens, Greece, entitled, "Treatment of Alveolar Pyorrhœa," was then read.

Passing over the symptoms and causes of this disease, the writer said he would speak only of its treatment.

From the point of view of the severity, I consider the malady as separable into four degrees, as facilitating the application of the treatment which I recommend.

I regard the disease as of the first degree when the suppuration has only extended to the neck of the tooth, the alveolo-dental periosteum being unaffected. The cure of this stage is extremely easy by the following treatment: In the first place, the tartar or other irritating deposits are to be completely removed; the gums are to be scarified as thoroughly as possible, with a steel instrument wrapped in cotton, which should as a preliminary be dipped in a 1 to 1000 solution of sublimate. This is at once followed by an application having the following composition:

Tincture of Iodine.

Tincture of Aconite, equal quantities.

The patient is directed to cleanse the mouth thrice daily with a brush and the following antiseptic lotion:

| | |
|---|---|
| Tincture of Thyme........................... | 2 grams. |
| " " Eucalyptus..... ............. | 1 gram. |
| " " Benzoin.......................... | 4 grams. |
| " " Mint ............................ | 120 grams. |
| " " Lavender........................ | 2 grams. |
| " " Rosemary........................ | 1 gram. |
| " " Cologne.......................... | 2 grams. |
| " " Anise ........ ...................... | 4 grams. |

Sig. One teaspoonful in half a glass of water.

Have the patient return at the end of a month; he will be entirely well if he has followed directions.

Second degree: Suppuration having extended to the upper portion of the cement. Treatment the same as for the first degree, only it will be necessary to have the patient return several times for scarification and painting of the gums. The cure will be complete at the end of two or three months.

Third degree: Suppuration involves the whole of the cement and the periosteum, but the teeth have not given way entirely. The visits must be more numerous, and the strength of the sublimate solution is to be increased to 2 to 1000. After scarifying the gums by means of a bistoury, I introduce with a Pravaz syringe the sublimate solution between the tooth and the gums, and at once apply the iodine and aconite preparation.

If there be inflammation and the patient suffers pain, after removing the tartar, I order emollients to relieve the pain before scarifying and employing the sublimate. Cure is not yet obtained in this degree of the malady. I continue the treatment until all suppuration has ceased, and the teeth have become almost immovable. It is possible to get these patients to masticate with comfort, who have had the severest suffering, and in whom the slightest pressure occasioned almost unbearable pain.

As to the fourth degree, which I define to exist when the teeth are altogether loose, the same treatment is to be employed, although a satisfactory result is rarely obtained. I have had some half-cures for a time, but it is only in persons with abundance of patience, usually the patient becomes wearied before experiencing the slightest improvement.

This paper was passed without discussion, and the Section adjourned to meet at 2:30 P. M., Thursday.

SECTION 5.—DENTAL AND ORAL SURGERY.

The section met at 2:30 P. M.

The Chairman, Dr. Brophy, spoke of his absence from the opening session of yesterday, and proceeded to deliver a brief opening address, sustaining the rights of the dental surgeon to perform any surgical operation pertaining to the oral cavity. He said: I hold that any dentist, educated within the schools of dentistry on the lines taught, is authorized under the laws of the State chartering the institution, to practice in either department and has equal rights to administer remedies and perform surgical operations with the general surgeon.

Dr. Crawford, of Nashville, was called to the chair, and a paper was read by Dr. Brophy on "the Operation of Staphylorraphy."

The principal difficulties encountered are those of tying the sutures, the great tendency of the sutures to slough out after they are once nicely secured, and the concealment of the parts during operation, both because of deficiency in light and the accumulation of the viscid muco-saliva, which in mouths thus affected is secreted in such abundance.

In my judgment it is sometimes best to divide the operation into two stages, making the first operation on the hard palate, and subsequently, after the process of repair is complete, an operation on the soft palate. If, however, the operation is to be made on both the hard and soft palates at the same time—and this is sometimes admissible—the edge throughout the entire length of the fissure should be first prepared.

His paper was a detailed description of a new operation devised by himself, to be performed in early infancy for the radical closure of the hard palate. This operation cannot be given in an abstract, every detail being essential to its full understanding.

Dr. W. C. Barrett said: I have known of the operation and have watched some of the cases, and I believe honestly and candidly that it is the most important and radical improvement in surgery that has been presented by a dentist. I believe it is something that is radical. I wonder that the world of surgery has not seen the point before. I have heard all sorts of objections urged against it. But I have had an opportunity to see a number of operations, and it does seem to me that it is marvelous.

I believe when a man has done something that is of value to the profession credit should be given to him, and I know that Dr. Brophy originated this operation.

Dr. Brewster: I think, with Dr. Barrett, that this is something which we will all appreciate more and more as time goes on, and the method of bringing those parts together is better understood.

Dr. Case: I have been aware that Dr. Brophy has been performing this operation for some time. I have seen him operate, and seen the result of the operation. It is certainly successful in every way, and is perhaps the very best method of closing the opening produced by a congenital cleft of the palate. This operation should be performed soon after the child is born.

Dr. Curtis: I would like to ask what success Dr. Brophy has had after the eruption of the teeth?

Dr. Brophy: The only difficulty is the breaking up of the antagonism of the teeth, and that is the reason why this operation should be performed while the child is young. That is one of the principal disadvantages in later life, that the occlusion of the teeth is broken up, and the operation is more difficult at a greater age, because the bone is harder and does not so easily yield.

Dr. Morgenthal: I took great pleasure in listening to the address of Dr. Brophy. I think the doctor ought to be congratulated upon devising a means to avoid the cutting of the palatal muscles. As an aurist, I commend very highly Dr. Brophy's operation.

Dr. Brophy: In closing the discussion said, I only desire to give the Congress and the individual members my thanks for the compliments they have so generously bestowed upon me.

The section then adjourned till 2:30 P. M. Thursday, August 17th.

SECTION 6.—OPERATIVE DENTISTRY.

This section was called to order at 2:30 P. M. by Dr. McQuillen, who presided.

Dr. Emil Schreier, of Vienna, Austria, read a paper on "The Treatment of Infected Root-Canals with Kalium-Natrium."

He said: I entertain the hope that the subject of my paper is of such importance for daily practice that it cannot fail to arouse the interest of a large proportion of those present. It is my purpose to lay before you a procedure for the antiseptic treatment of root-canals, which from its great simplicity and ease of application, as well as on account of the many excellent results which have been obtained therewith, deserves consideration from this distinguished assembly. I refer to the method of treatment introduced by me with kalium-natrium (potassium and sodium).

When a tooth with gangrenous or necrotic pulp comes under treatment, the dentist is confronted with the task of removing as far as possible a gelatinous, slightly consistent mass from a capillary tube, and this having been accomplished, to introduce into the same canal an antiseptic for purposes of disinfection. You are all aware how much time, patience, and skill are necessary for this operation, and will readily recognize that the operation would be much simplified if it could be made possible to effect the transformation by the simple introduction of a nerve-needle.

My method seeks to fill the first indication by a chemical decomposition of the putrescent contents, in which the root-canal serves as a test-tube; the second indication is fulfilled in the development of a substance which is readily taken up by a nerve-needle, and sufficiently adhesive for introduction into the canal.

In the root-canal in question there exists a putrescent mass. This consists of water and the decomposition product of albumen, the latter consisting especially of fats and fatty acids.

These substances have been formed by the influence of bacteria, and serve as a culture-medium for the various species contained therein. If I now introduce my preparation into the canal with the needle, decomposition of the watery contents will occur, with development of a considerable amount of heat. Potassium and sodium hydroxids are formed, which, in combination with the fat of the pulp, form soap. The characteristic gangrenous odor is accordingly changed into a well-marked soapy smell. A portion of the alkalies possess the well-known property of rendering albuminous substances soluble. Thus, any remains of tissue adherent to the walls of the canal are dissolved. The latter become lacerated, and access to the dentine canaliculi is possible sooner than can be effected by any other method thus far employed. Destruction of the organic contents of these canals is now possible. You will readily understand that in consequence of such destruction the disagreeable discoloration, which too frequently occurs, will be absent, and that the lime-salts of the tooth proper are in no wise injuriously affected by the treatment.

The introduction of the potassium and sodium has the additional effect of destroying the bacteria, partly by the heat produced, and partly by the new products formed. The contents of the canal have been transformed into a sterile and probably antiseptic mass, and thus the development of the new colonies of bacteria is prevented. Everything has thus been accomplished which precedes permanent filling of the tooth.

I hope to have the opportunity of demonstrating my method on the living subject, and you will see how the transmitted mass travels in the direction of least resistance—that is, into the orifice of the canal next the pulp-chamber, and wells up alongside of the needle. It is scarcely necessary for me to state that my plan of treatment should only be practiced with the coffer-dam. Of course, care must be exercised in manipulating with the preparation. With proper care the preparation is free from all danger. I believe that I am entitled to say that the plan of treatment proposed by me is founded upon correct principles, and meets the obvious indications as regards ease and rapidity of application and certainty of result. Every practitioner is in a position by the aid of my preparation to save with rapidity, ease, and well-nigh with certainty, teeth that have been seriously affected, and that without special preliminary preparation, and without troublesome appliances. Thus the benefit of treatment of the root becomes possible for the masses.

In the discussion which followed, the question was asked: Can you regulate the heat by the strength of the preparation which you use?

Dr. Schreier: No, not at all. I can only regulate it by taking more or less of the preparation. If you take too much of the preparation it is dangerous.

Dr. Custer: How do you wash the canals after using the preparation?

Dr. Schreier: After using the preparation the canal is filled with a soapy matter as the result of the chemical action. I wash the canals with water, or I take some weak solution of carbolic acid and water, so as to be sure not to carry into the canal any bacteria. For getting out all the particles of soapy matter, I wrap a few fibers of cotton around the broach, dampen it with water, and then revolve it rapidly in the canal because the soapy matter is soluble in water. This procedure will quickly clean the canal.

Dr. A. H. Brockway, Brooklyn, N. Y.: I have had a little experience with this preparation, and I suppose that experience will be valuable to those who have not used it. Some weeks ago I got the preparation and have used it in quite a number of cases, and I must say I am extremely pleased with the results so far.

Dr. Reese: I have used the preparation since May, in about a dozen cases, and the patient did not know anything about the heat developed by the chemical action. In removing the soapy contents of the canal I have used peroxide of hydrogen; after drying the canal with cotton, put in the treatment with some oil and leave it there for a week, and then I find the canals have a cleaner feeling than by using any other method.

Dr. Reid: What is the proportion of sodium and potassium?

Dr. Schreier: It is not a fixed quantity, but usually I use two parts of sodium and one of potassium, prepared in such a manner that it will adhere to the nerve-broach.

Dr. W. B. Ames, of Chicago, Ill.: I would like to know in what sort of cases this preparation would be used. Would it be used in a case when the pulp had been devitalized a day or two, or when the pulp was devitalized two or three weeks ago?

Dr. Schreier: I treat it immediately after destroying the pulp if there is insensibility. Any hemorrhage will be stopped by the introduction of the preparation.

Dr. Ames: Would this combination of sodium and potassium act the same on the blood as the pulp?

Dr. Schreier: It would.

Dr. Ames: I want to know whether in Dr. Schreier's experience there is much after inflammation in a few days?

Dr. Schreier: No. There is no soreness following the application. If a patient presents himself with a sore tooth I proceed in the same manner as I do with the others.

Dr. Arnold, Columbus, Ohio: Would Dr. Schreier's method of treatment be different from what he has explained if there had been absorption in the apex of the tooth, or if a blind abscess were present?

Dr. Schreier: I don't make any change in the treatment; I always find the fistulous openings closing after three or four days.

Dr. Florestan Aguilar, Cadiz, Spain: I received a sample of it last September. I have used this preparation in all sorts of cases, where the pulp had been devitalized for a long time, and also in cases where the pulp has been devitalized only for two or three days; and I have used it in teeth which had abscesses, and my experience has almost always been satisfactory. In a very few cases I have met with failure, but I did not blame the preparation; I think everybody has some failures in treating root-canals. I am very much pleased with the result of this preparation of sodium and potassium. I have gone a little further than Dr. Schreier in the employment of this preparation; he says he waits two or three days. I have had as good results by filling the canals immediately after cleaning them. I am always very careful in removing the rubber-dam, because if you let a little drop of the preparation fall on the cheek or gums it is apt to leave a mark on the face, which is very painful to the patient.

Dr. W. B. Ames, Chicago, Ill., next read a paper on "Oxyphosphates."

He said, that in the present study he would consider the oxyphosphate cements from a physical rather than a chemical standpoint, for the reason that there is less difference chemically than in their physical characteristics.

In all the cements known to him, with two exceptions, the acid is intended to be either pure ortho-phosphoric or an ortho-phosphoric acid solution of alkaline phosphates. Extensive endeavors to utilize pyro-phosphoric acid for the purpose had led him to consider it impractical and unreliable. An occasional specimen will be all that could be desired, but the impossibility of duplicating it with any degree of exactness precludes any reliance on this variety of acid.

The alkaline phosphates are all soluble in water, and these are the only phosphates that will remain in solution in phosphoric acid for any considerable time. All metallic phosphates in such solution will in time re-crystallize, partly or wholly, which precludes their use for the purpose of modifying the working properties of cements. The object accomplished by the addition of phosphates to phosphoric acid is, in cement, to retard the setting and render it less caustic.

Among cement powders, there is less difference chemically than among the liquids, but physically they differ very radically. The basis of all light-colored cement powders is oxide of zinc. This material can exist under many physical conditions, and yet be simply ZnO.

He expressed the belief that a thoroughly crystalline zinc oxide is much better adapted to the requirements of an ideal oxyphosphate cement than the vitrified amorphous oxide, and gave the results of using the liquids and powders of various cements conjointly, as follows:

I have found that I can get better results by using a crystalline oxide with many of the adulterated liquids than by using the oxide that is furnished with them. Then with the group of cements in which unadulterated ortho-phosphoric acid is furnished I find that I can use the powders and liquids of all conjointly, getting almost any working quality desired. For instance: the powder of the Harvard is thoroughly crystalline, and of a very fine state of division. With this powder and the liquid of Justi's Lapidescent, I have a better cement for crown or inlay setting than by using either of these cements as furnished. This combination can be worked quite stiff, and yet have a smooth plasticity that facilitates the operation with satisfactory setting qualities. The powder of the Lapidescent is less crystalline than that of the Harvard, and the liquid of the Harvard does not give as satisfactory hardening as some of the others of its type. The combination of Harvard liquid and Ames powder gives more satisfactory crystallization than Harvard entire. The Ames liquid and Harvard powder works nicely, does not set as quickly as Ames, but much quicker than Harvard.

The lapidescent liquid and Ames powder makes a good mixture, setting a little quicker than lapidescent liquid and Harvard powder. For fillings, where great resistance against wear is required, I have nothing at my command in which I have more faith than the Ames crystalline, having a quick and medium slow variety at my command. A more definite crystallization takes place in its hardening than with most other cements. The surface will take on a glassy appearance in the mouth that gives promise of great wearing qualities. It also comes nearer being a submarine or hydraulic cement than those which are termed "hydraulic," and yet are furnished with the caution to "have the cavity perfectly dry and keep on the rubber until thoroughly hard."

Both the paper and the discussion rather limited the value of the cements to their use in crown and bridgework.

Dr. McKellops said: I am a little astonished that the gentleman who read the paper did not allude to oxyphosphate as a filling material. I look upon oxyphosphate as a great thing in filling children's teeth or teeth of persons where gold work will not stand. Take a young child, and you can nurse its teeth with oxyphosphates until a time when you can fill with gold, and you will not find any decay where oxyphosphate filling is put in properly.

I can save more teeth in children by the use of oxyphosphates where everything else fails. The fillings wear out, some in one year, some in three or four or five, but they can be replaced. I believe I have one in my mouth that has been there twenty years.

Dr. J. Taft, Cincinnati, O.: In using oxyphosphates three or four things must be taken into account. The quality of the material itself is important, another is the manner in which it is manipulated; because, in many instances, a very good material proves totally insufficient by reason of the defective method of using it. Another point that must be taken into account is the position where it is to be placed. It is subject to disintegration where it is exposed to two destroying influences; one, mechanical force, another, the solvent power of the fluids of the mouth. The manipulation of the material on a cold slab is an important point. If the temperature is right, there is no occasion for cooling the slab, but the material should be manipulated correctly. In some cases it should be of greater consistency than others. In crown-work you cannot use the same consistency as in an exposed cavity.

I have sometimes used a little slab of porcelain and cemented it in a cavity of children's and adults' teeth, and it has worn for years. It prevents the wearing of the masticating surfaces of the molars, and the cement below will be protected from the solvent fluids of the mouth. I regard it as almost criminal to take a child of ten, twelve, or fourteen years of age, when the teeth are decaying rapidly, and make long, tedious operations in gold, when the work will be of a mere temporary character.

A paper by Dr. D. Caracatsanis, of Athens, Greece, was read, as follows: "On the Treatment of Dental Caries in the Second, Third and Fourth Degrees."

He spoke of the importance of temporary fillings in these cases, and said: For the last four years I have proceeded in the following manner to my entire satisfaction.

Second degree: Cavity is cleansed, cauterized with absolute alcohol, and immediately filled. If the dentine is very sensitive, I coat the cavity (after well washing and drying) with a liquid preparation of gutta-percha dissolved in chloroform or oil of cajuput. I then at once fill the cavity with simple gutta-percha, which I leave in place for eight days. If the sensitiveness has disappeared at the end of that time, I put in the permanent filling. If the contrary is the case, I remove the gutta-percha and repeat the operation, which procedure may be repeated until the fourth time, for cases with extreme sensibility.

Third degree: Remove the carious margin, take away the remains of food and softened dentine, carefully wash the cavity with a warm antiseptic solution, and employ the following two preparations:

| | | |
|---|---|---|
| 1. Oil of Cloves | 3 | grams. |
| Oil of Peppermint | 2 | " |
| Chloroform | 5 | " |
| Acid Phenique | 1 | " |
| Collodion | 2 | " |
| Cocaine | 1 | " |

| | | |
|---|---|---|
| 2. Metallic Cobalt | 5 | parts. |
| Arsenious Acid | 1 | part. |

Saturate a cotton tampon with the first preparation and then with the second, place at the orifice of the pulp without pressure, cover with a dressing of cotton in order to protect the neighboring parts and those cauterized. These dressings will have to be changed several times before the nerve is completely destroyed. Their action is slow, but painless. Then carefully wash the canals. If these are very small I don't try to open them—a useless attempt, since it is never possible to do so as far as the apex, and, moreover, there is risk of perforating the root. After carefully washing and drying the cavity, I introduce by means of an insufflator some powdered boracic acid. At the end of eight days, if everything goes well, I remove the temporary filling and fill the opening of the canals as far as possible with liquid gutta-percha and also the nerve canals. If the canals are large, the liquid may be introduced either by the aid of a tampon or by means of a little syringe. The gutta-percha is covered and also left in its place for eight days. If, during this time, inflammation occurs, I remove the temporary fillings and re-commence treatment until the cure is complete.

I never fill a tooth of the third degree even if the canals are widely open, before introducing, after the destruction of the pulp, dressings consisting of aromatic oils.

For teeth of the fourth degree, whether with alveolar abscess or not, I employ all the disinfectants and antiseptics, I never give up all hope of filling until a number of sittings for the practice of complete antisepsis. On first filling, in place of boracic acid, I introduce iodoform by means of an insufflator. Temporary fillings are especially valuable in this degree.

Conclusion: According to my experience and statistics, the following class of cases should never be permanently filled.

First, in a tooth of the second degree, the dentine of which is too sensitive, the filling with the chloroform base is the best therapeutic.

Second, the best possibility is in retaining the pulp in the teeth of the third degree, and the necessity of putting the teeth to the test of a temporary filling. A tooth of the fourth degree cannot be disinfected and filled at one sitting, and temporary filling is above all indispensable for this degree.

On motion, the meeting here adjourned to Thursday.

SECTION 7.—PROSTHESIS AND ORTHODONTIA.

The Chairman, Dr. C. L. Goddard, called the Section to order at 2:30 P. M.

The discussion of the paper read at the previous meeting by Dr. V. H. Jackson, entitled, "A Method of Constructing Spring Appliances for Correcting Irregularities of the Teeth," was continued.

Dr. A. E. Matteson thought that in a number of Dr. Jackson's cases the effect would have been more easily attained by the use of a jackscrew. He thought the jackscrew, in appropriate cases, to be preferred over any other appliance.

He continued: Dr. Jackson, in his paper, says nothing about what he considers the best age in which to begin the process of regulating teeth. His opinion was that the earlier the better. The deciduous molars are more firmly fixed than any other tooth in the mouth, and they can safely be employed as points of resistance to direct the movement of the erupting anterior teeth if they are out of line, especially as it takes very little force to direct their growth.

Dr. Jackson closed the discussion. He said: That Dr. Case had said that the action of the screw was more near to the action of nature than the constant action of a spring. He supposed he referred to the law of nature recognized by gymnasts and athletes, that action succeeded by rest causes increased growth.

In our case increased growth was not the desideratum aimed at. We want absorption, and this is produced by continual pressure. Both experience and theory will uphold the continued action of springs as the best force to move teeth in regulating.

Discussion closed, and Dr. D. Caracatsanis read the following paper: "On the Possibility of Avoiding Metallic Clasps in Partial Dentures of Vulcanite."

According to the method of the writer almost any small plate may be kept in place without any clasp.

He said: I place a single incisor in the upper jaw with the greatest ease with a plate of small dimensions and without any clasp, by means of white caoutchouc. To insert the teeth, I take advantage of a vacant space, produced by removal of a tooth or of a natural space between two teeth for fastening the extremities of the caoutchouc, instead of using clasps. With points of support which have seemed insignificant I have been able to anchor a denture of eight or ten teeth very solidly, the plate being the width of a centimeter or a centimeter and a half at most.

I have seen abuses of the clasp method, as, for instance, of a partial upper denture carrying by means of metal clasps the second right bicuspid, the right lateral incisor, the left central incisor, left cuspid and the second left bicuspid. At the end of a year the teeth were entirely loosened, the tooth structure worn away by the friction of the metal, and the patient suffered intense pain in the teeth from changes of temperature. I replaced the piece with one without a clasp, taking care to avoid the slightest pressure on the loosened teeth, and securing the re'ention of the denture in place solely by rubber clasps, affixed as the preceding ones had been to the first molars. The piece kept its place as well as its predecessor, and the plate was no larger. The advantages to be derived from this plan, which concern not only the patient but the dentist, are economy of material, since metal is avoided; economy of time, and the facility of making a light plate which causes no inconvenience for the patient.

The fact that Dr. Caracatsanis could not understand English, and very few of those present understood any French, made the discussion very difficult; but the author of the paper said, that he would give an opportunity for all to get an understanding tomorrow at the clinic, when he would make a plate such as he had described.

The President called the Vice-President to the chair and read the following paper, "Separation of the Superior Maxilla at the Symphysis."

The author described a case in which in spreading the arch such a separation had obviously taken place, and said: No pain or discomfort accompanied or followed the case I have described. I have kept the arch spread, thinking that a deposit of osseous material in the suture would help to retain the exact width of arch that I have gained, and give the room needed for correcting the irregularity of the incisors. The centrals were rotated and the laterals drawn forward till they had assumed their proper positions.

Following the case described, the author gave a detailed description of appliances which he had devised and found useful for spreading the arch. He regarded the commonly-accepted explanation that the changes consisted in absorption of tissues in front of the moving teeth and building up behind, as not satisfactory, for he had in many cases observed a movement of the alveolar process itself.

Dr. Goddard further called attention to the great importance of proper instruction in orthodontia in our dental schools. It should be taught practically as well as didactically.

In the discussion of this paper Dr. H. R. Staley said, he objected to Dr. Goddard's manner of regulating, because it did not move the tooth bodily but only moved the crown. He thought that the operation should be such that the tooth would be moved, roots and all, and not merely tipped in the direction it was desired to take.

Dr. Harcourt gave an instance in his practice of the division of the arch at the symphysis.

Dr. E. S. Talbot said, as to the opening of the suture, he had frequently done it. If the correction of an irregular denture is attempted before the age of twelve or fourteen years, it is very commonly done. In the case of his own daughter the sutures opened about three-sixteenths of an inch, and because it opened

I was able to accomplish as much in correcting the arch in two weeks as I had expected to do in three months. He is convinced that the jaw bends; he did not believe it formerly. Dr. Farrar says it does, but now he knows that it does. However, in cases where space is gained by the opening of the suture the centrals do move in and fill the space again, but it is because of the force excited by the forward pressure of the other teeth.

Dr. Staley asked how long it would be necessary to retain the appliance after the opening of the suture, so as to give the bone time to form before the pressure is allowed to come back on it.

Dr. Goddard said, that there was no question that the bone would fill up, before it would be possible to gain the space needed between the teeth. He had never been able to move the roots of teeth to any great extent. Sometimes it would be very desirable to do this, especially when in an incisor where the roots were considerably inside the line, for if the crown should be moved to line the projection would be so disfiguring that he thought it better to extract such a tooth and supply an artificial substitute.

SECTION 8.—EDUCATION, LEGISLATION, AND LITERATURE.

Called to order by Dr. J. J. R. Patrick, chairman, at 2:30 P. M.

The report of the Committee on Dental Nomenclature was read by Dr. G. V. Black.

He said: The task assigned to this committee to "present a plan by which a universal system of nomenclature may be adopted by the Congress that would be acceptable to the profession of the entire world," is an exceedingly difficult one.

The nomenclature of any science or art is a growth, and it has been necessary to trace the steps of the growth of dental science in order to understand the formation of its nomenclature, and gain a clear conception of its future tendencies. We cannot sit down and construct a nomenclature for dentists any more than we can arbitrarily prescribe the words to be used by any other class of people.

Your reporter has found no recognized rules of dental nomenclature that will serve as a basis upon which to proceed to the improvement of existing forms. We are therefore without a basis of action until such a scheme is made known and recognized in such a way as to form the basis of discussion.

The necessity for a definite system was pointed out, and an elaborate scheme, as a basis for further work by the committee, laid down. This does not admit of abridgement, and is too lengthy to be given here as a whole.

There was no discussion.

Dr. Garrett Newkirk, Chicago, read a paper entitled: "Nomenclature Relating to Forms of the Dental Arch and Special Positions of the Teeth."

This paper was illustrated by diagrams, the dental arch being typified by the base and apex of the ellipse, as the egg and the leaf.

The author criticised the commonly-accepted terms, and in describing the dental arch as well-rounded, angular, full elliptical, V-shaped, saddle-shaped, all of which terms are indefinite or misleading, if not entirely unscientific. A dental arch cannot be round, neither does it ever describe a segment of the circle. "Angular" means nothing definite, unless we are told the number, location, and forms of the angles. The great majority of the arches are elliptical, but ellipses vary extremely. We have no accepted way of qualifying the term elliptical, and it has the disadvantage of being a word with four syllables.

May we not in this connection borrow a few hints from botanical terms descriptive of the forms of leaves? For example, the word *ovate*, derived from *ova*, the egg. It is represented by the longitudinal section of an egg.

Strange to say, the egg lines, when we come to analyze them, represent two forms of the ellipse combined.

The base of the egg, as we will say, is outlined by the segment of a short ellipse, the apex by the segment of an ellipse nearly twice as long. We have, therefore, in the egg-form term something which would answer to the outline of the dental arch in a very large majority of cases.

From *ova*, the egg, we would have *oval*, *ovate*, or *ovoid*. The V-shaped arch is simply *ap-ovoid*. Where the central incisors are so placed as to suggest an angle we would have the word *conoid.* The "Saddle-arch" is a constricted *ovoid.*

Where the line of the incisors is nearly straight from cuspid to cuspid we have the descriptive word *truncoid*, etc., etc.

As to the position of the teeth relative to the arch, which are said to be "outside" or "inside" of the arch, the four terms: *protrusal, intrusal, extrusal*, and *subtrusal.* From these we could compound the terms: *Mesio-protrusal, Disto-intrusal*, etc.

For a rotary displacement he suggests the word *tort* "a condition twisted," or "it was tortal," the force used to correct this malposition being *torsion.*

Dr. Patrick called Dr. Newkirk to the chair, and opened the discussion, which was continued by Drs. Crawford, C. R. Taylor, and Noyes. Dr. Black concluded the discussion, saying: "We may fix nomenclature till we are gray, and until the next generation grows gray, but there is no fixity while science is progressive."

Adjourned to 2:30 P. M., Thursday.

